# Physicians' attitudes about artificial feeding in older patients with severe cognitive impairment in Japan: a qualitative study

**DOI:** 10.1186/1471-2318-7-22

**Published:** 2007-08-17

**Authors:** Kaoruko Aita, Miyako Takahashi, Hiroaki Miyata, Ichiro Kai, Thomas E Finucane

**Affiliations:** 1 Department of Social Gerontology, School of Health Sciences and Nursing, The University of Tokyo, 7-3-1 Hongo, Bunkyo-ku, Tokyo 113-0033, Japan; 2 Department of Healthcare Quality Assessment, School of Medicine, The University of Tokyo, Japan; 3 Division of Gerontology and Geriatric Medicine, School of Medicine, The Johns Hopkins University, Baltimore, Maryland, USA

## Abstract

**Background:**

The question of whether to withhold artificial nutrition and hydration (ANH) from severely cognitively impaired older adults has remained nearly unexplored in Japan, where provision of ANH is considered standard care. The objective of this study was to identify and analyze factors related to the decision to provide ANH through percutaneous endoscopic gastrostomy (PEG) in older Japanese adults with severe cognitive impairment.

**Methods:**

Retrospective, in-depth interviews with thirty physicians experienced in the care of older, bed-ridden, non-communicative patients with severe cognitive impairment. Interview content included questions about factors influencing the decision to provide or withhold ANH, concerns and dilemmas concerning ANH and the choice of PEG feeding as an ANH method. The process of data collection and analysis followed the Grounded Theory approach.

**Results:**

Data analysis identified five factors that influence Japanese physicians' decision to provide ANH through PEG tubes: (1) the national health insurance system that allows elderly patients to become long-term hospital in-patients; (2) legal barriers with regard to limiting treatment, including the risk of prosecution; (3) emotional barriers, especially abhorrence of death by 'starvation'; (4) cultural values that promote family-oriented end-of-life decision making; and (5) reimbursement-related factors involved in the choice of PEG. However, a small number of physicians did offer patients' families the option of withholding ANH. These physicians shared certain characteristics, such as a different perception of ANH and repeated communication with families concerning end-of-life care. These qualities were found to reduce some of the effects of the factors that favor provision of ANH.

**Conclusion:**

The framework of Japan's medical-legal system unintentionally provides many physicians an incentive to routinely offer ANH for this patient group through PEG tubes. It seems apparent that end-of-life education should be provided to medical providers in Japan to change the automatic assumption that ANH must be provided.

## Background

Percutaneous endoscopic gastrostomy (PEG) tube feeding has caused great controversy in some western countries [1,2]. Clinical evidence supporting the use of tube feeding in patients with advanced dementia is limited [3-8], yet PEG utilization remains quite high [9,10]. PEG feeding is expected to become even more controversial in Japan where, in contrast to other developed countries in the West, provision of artificial nutrition and hydration (ANH) remains a social norm [11]. Previous studies in Japan have shown that PEG is used most commonly for bed-bound, incapacitated patients over age 75 who have cerebrovascular disease or dementia [12-15]. Some Japanese physicians and nurses are vocal critics of the routine provision of ANH without regard for the patient's condition [16-18]. In one survey, a great majority of the Japanese population expressed an unwillingness to be fed through PEG tubes if they become bed-bound, and are unable to eat or communicate [19]. Nonetheless, Japanese physicians usually claim that they have no choice but to provide ANH to such patients [20]. The purpose of this study is to understand why Japanese physicians feel bound to provide ANH, particularly through the use of PEG tubes, to older patients with severe, irreversible cognitive impairment. This study focuses on factors that encourage physicians to use PEG tube feeding in this patient group.

### The national health insurance system in Japan

To describe financial incentives in Japan, an understanding of the health insurance scheme is needed. Universal health care was introduced in 1961, and since then, has aimed to provide all citizens with equal access to health care at reasonable cost. In addition, individuals over age 75 receive additional financial assistance under a separate old-age healthcare system. Due to a lack of good alternatives, hospitals have often assumed the function of nursing homes for the frail elderly [21]. Because Japan's population has been aging steadily, medical costs have been rising towards a major financial crisis in recent years [22]. The government responded incrementally. In 1990 a new system of inclusive per-diem payments was introduced as an option for hospitals that had high percentages of geriatric patients [23]. The measure was gradually strengthened until 2003 when the government completed its national classification of hospitals into either acute care hospitals, which are basically run under the fee-for-service system, or long-term care hospitals, which are run under an inclusive per-diem payment system. This classification of hospitals was intended to discourage physicians, particularly those at privately-owned hospitals, from utilizing expensive procedures that had been encouraged by the fee-for-service system. As of early 2006, there were about 380,000 beds in long-term care hospitals across the nation, most of which functioned as de facto nursing homes. These cost about 440,000 yen (~ US$3,660) or 490,000 yen (~US$4,260) per month per bed depending on the insurance plan [24]. For a patient over age 75, co-payment was required and depended on income; for a patient with an average income, monthly co-payment was 40,200 yen (~ US$335).

## Methods

We conducted a qualitative, exploratory study using in-depth interviews with physicians who care for elderly patients with severe cognitive impairment because of advanced dementia and who have participated in decisions regarding the use of ANH. The process of data collection and analysis followed the Grounded Theory approach, which is a methodology to inductively develop a theory from data, systematically gathered and analyzed through the research process [25]. The methodology originated from a sociological monograph about dying in 1960s [26].

### Recruitment of informants and data collection

Purposive sampling was used to identify internists and surgeons from certain specialties, including neurology, neurosurgery, gastrointestinal medicine, and gastrointestinal surgery. Because geriatrics is not a well-recognized specialty in Japan, physicians at long-term care hospitals usually do not identify themselves as geriatricians, even though they deal primarily with frail older adults. This sampling aimed to gain interview data representative of the various specialties that care for patients who might need PEG feeding. Similarly, attempts were made to include physicians and surgeons of different ages and from different types of hospitals in terms of operator, classification and size of the institution to compare their similarities and differences. All informants were actively involved in the care of elderly patients with severe cognitive impairment. Concurrently, theoretical sampling [25] was also conducted, whereby informant selection was guided by ongoing analysis. In particular, during a later phase of the study, we searched for doctors who routinely offered patients' families the option of withholding ANH, because we found that very few Japanese doctors do so. Additional interviews were conducted until theoretical saturation was reached, that is, until no new themes or data were elicited [25]. This occurred when 30 informants had been interviewed. Theoretical saturation is a point when finalizing the data-gathering process because no new or relevant data are able to be further obtained.

Interviews were conducted mainly in the Tokyo metropolitan area from February to October 2004. All interviews except one were recorded and transcribed verbatim. Written informed consent was obtained from each informant. The single informant who declined to be recorded did give permission for notes to be taken during the interview and for a rough transcript to be generated within the day of the interview. The interviews ranged from half an hour to two hours in length (average of one hour per informant). All interviews were conducted by the principal investigator, and most interviews took place in a quiet environment at the informants' workplace, such as in a counseling or conference room. Interviews began with questions about the informants' demographic background, followed by questions on decisions about ANH in the informants' clinical experience. The following issues were assessed: information provided to patients' families prior to decision-making and how this information was delivered, factors related to either providing or withholding ANH, ANH-related problems, informant's concerns and dilemmas concerning ANH, and whether the informant would want ANH for themselves if he/she were in the same position as his/her patients. The rough framework of the interview questions was pre-determined, and open-ended questions to follow-up details and unexpected responses were added during each interview, depending on informants' comments. In an effort to ensure informants' privacy, all interview data which included confidential information were transcribed by the principal author. In addition, informants were assured that information would be collected and maintained confidentially. Identifying details were removed from the transcripts and filed separately in a locked filing cabinet in the principal author's office.

### Analysis

Analysis was done as transcripts became available. In the open-coding process, transcripts were reviewed line by line and conceptual labels were attached; similar concepts were then grouped together to form categories. Concurrently, the relationships between the categories were examined in a process called axial-coding, where the 'axis' of a category is defined to determine its relation to its subcategories. The authors examined primary data, and emerging open and axial codes to generate a working theoretical framework, which was then iteratively re-compared to both the data and codes. In the selective-coding stage, categories were integrated to form a larger explanatory model. Open-, axial-, and selective-coding processes were accompanied by regular meetings among the authors during which they examined the transcripts, emerging concepts, categories, working hypotheses and theoretical framework in a sequential and iterative process. To enhance credibility of the analysis, member checks [27] were conducted, i.e., the authors sent the preliminary analysis report to three informants for feedback. One of the three practiced at an acute care hospital, while the other two were from long-term care hospitals. Of the latter two, one routinely offered patients families an option of withholding ANH, while the other had a strong belief that ANH should never be withheld. All three informants replied that the analysis adequately explained the circumstances surrounding this issue.

## Results

The informants consisted of 25 men and 5 women aged 26 to 70 years, with a mean age of 47 (Table 1). The percentage of female physicians in this study was comparable to that of the national statistics compiled by the government [28]. Data suggested that for bed-bound, incapacitated elderly patients, decisions about PEG tube placement occurred primarily in two contexts: in acute care hospitals during acute illnesses, especially severe, disabling stroke, and in long-term care hospitals when dementia led gradually to difficulty in eating and swallowing. Data analysis identified a total of five factors which favor a decision to initiate PEG tube feeding. The first factor is related to the overall clinical environment, in which the national health insurance system allows elderly patients to become long-term hospital in-patients. Three factors deter physicians from offering the option of withholding ANH: the legal consequences of limiting treatment that might be life-sustaining; the physicians' emotional reluctance to allow death without clearly providing food and water; and cultural values that promote family-oriented end-of-life decision making. Finally, the current reimbursement system provides incentives favoring PEG tube instead of other ANH methods. This research also found a small number of physicians who do offer families the option of withholding ANH and who share certain characteristics. Representative quotations are presented below (No. refers to the informant's ID number).

**Table 1:** Characteristics of informants

No.*	**Age**	**Sex**	**Hospital classification ^**†**^**	**Specialty ^**‡**^**	**No.**	**Age**	**Sex**	**Hospital classification**	**Specialty**
**1**	35	M	Acute	Internal medicine	**16**	32	F	Mixed care	Psychiatry
**2**	30	M	Acute	Internal medicine	**17**	50	F	Mixed care	Internal medicine
**3**	37	M	Acute	Surgery	**18**	60	M	Long-term	Psychiatry
**4**	55	M	Acute	Internal medicine	**19**	45	M	Acute	Neurology
**5**	70	M	Acute	Neurology	**20**	48	M	Acute	Neurosurgery
**6**	47	M	Acute	Neurosurgery	**21**	44	M	Acute	Geriatrics
**7**	63	F	PCU	Palliative care	**22**	26	F	Acute	Internal medicine
**8**	51	M	Long-term	Internal medicine	**23**	47	M	Acute	Internal medicine
**9**	48	M	Acute	Neurology	**24**	45	M	Acute	Gastrointestinal surgery
**10**	42	M	Acute	Neurology	**25**	62	M	Long-term	Psychiatry
**11**	37	M	Mixed care	Internal medicine	**26**	35	M	Acute	Gastrointestinal internal medicine
**12**	52	M	Long-term	Surgery	**27**	50	M	Home care	Family physician
**13**	46	M	Long-term	Internal medicine	**28**	48	F	Home care	Family physician
**14**	52	M	Long-term	Neurosurgery	**29**	50	M	Mixed care	Internal medicine
**15**	34	M	Mixed care	Internal medicine	**30**	43	M	Long-term	General medicine

* Each informant was given an ID number.

^†^In the hospital classification column, mixed care means a hospital that has both acute care and long-term care wards.

^‡^Physicians at long-term care hospitals usually work as geriatricians as well.

### Overall clinical environment

The informants pointed out that Japan's universal health insurance system, in which elderly patients are given additional financial support, enabled many elderly patients to become long-term hospital in-patients at an affordable cost. Physician informants said that they usually do not consider the serious financial situation of the nation's health insurance system in their life-sustaining treatment decisions, including ANH, and they do not believe that lay people do either. As Informant No.9 stated, "I think the distinctive feature of end-of-life issues in Japan is that we do not talk about them from the perspective of healthcare economics. Financial matters are something that we should discuss openly, but in fact we actually seldom do. Providing patients who have no hope for recovery with life-prolonging measures means that they are financially supported by somebody else. However, Japanese people do not like to talk about these things. Not only lay people, my colleague physicians and I myself don't even think about this either in our daily practice." Under the circumstances mentioned above, physicians can offer patient families the option of ANH without discussing, and perhaps without considering, the financial implications.

### Factors that promote routine use of PEG tube feeding

Physician informants describe extreme difficulty in offering patients' families the option of withholding ANH, and refer to the following three factors: legal barriers, emotional barriers and cultural values. Choosing PEG tubes as the method to deliver ANH was partly attributed to reimbursement issues.

#### Legal barriers

Most informants believed that ANH is standard care, and that if they did not initiate ANH they would risk either litigation from patients' families or criminal prosecution, because Japanese law does not guarantee protection for withholding or withdrawing life-sustaining treatment. Informant No.10 stated, "There is no system that protects physicians who withhold ANH. Besides, there is no social consensus on the issue. Under these circumstances, I believe we have no choice but to give ANH to a patient who cannot eat." Another legal barrier is related to the lack of a proxy decision-making system. Physicians in Japan have virtually no way of learning the preferences of formerly competent patients because there is no policy in place for this purpose. Problems arise when treatment-limiting decisions have to be made by relatives on behalf of an incompetent patient. If relatives do not come to an agreement, physicians tend to follow a conservative option, and thus, provide ANH.

#### Emotional barriers

Informants described several personal emotions that made it difficult for them to withhold ANH: the abhorrence of 'death by starvation', the gravity of the decision to allow death to occur, and the difficulty of 'doing nothing' for a patient. Among these, abhorrence of 'death by starvation' was dominant. Most informants stated that they believe that the provision of ANH is inherently necessary for anyone who cannot take food and fluids orally. These informants contended that ANH is a basic, indispensable form of care, and they believed that the patients' families shared this view. Demonstrating this opinion, Informant No.20 stated, "Withholding ANH constitutes an act of abuse. You know it directly leads to death and this is the same as letting babies die without giving food." Informants also discussed the fact that 'doing nothing' for a patient and allowing a natural death would bring about more stress to physicians than providing potentially life-prolonging measures would. Informant No.2 stated, "Withholding ANH from a patient would make me feel as if I did nothing, but just stood by and watched a patient die". Some informants said it is partly attributable to a long-held medical primacy of prolonging and saving life at any cost. As Informant No.19 stated, "Physicians have been taught to prolong life anyway. It is always a problem knowing when to withhold treatment in the absence of guidelines. In addition, I believe physicians would experience a lot of emotional distress if they withheld care."

#### Cultural values

Informants also placed considerable importance on continuing the life of a patient for the family rather than for the patient himself/herself. Informants often said the main purpose for prolonging life with ANH was to meet the expectations of the family who, they believe, want the patient to continue living. Informant No.12 stated, "It is not the patient but the family who wants to have PEG performed on the patient. The patient is incompetent and has no choice but to be kept alive because the family wants the patient to be alive. Most patients with PEG tubes at my hospital belong to this category." Some informants said that in Japan, patients in a vegetative or minimally conscious state are kept alive not for themselves, but for the sake of the family. Reflecting this view, Informant No.25 said, "In a sense, Japanese people have to live not to fulfill their own happiness but their families' when they fall into this situation. I believe that is the most significant characteristic that makes Japanese families different from those in the West." The informants also observed that even a patient who is non-communicative remains the same person who had lived a meaningful life with the family, and hence, the patient's existence bolsters the family emotionally. As Informant No.13 stated, "A patient is just lying in a hospital bed, but I have noticed that in some cases, the patient's existence is of significant value for the family, uniting other family members." Also, informants felt unprepared to deal with the common situation in which relatives who rarely came to see a patient appeared at the last minute to demand more medical care. They observed that these relatives made claims not for the patient but for their own emotional needs.

From a different perspective, some informants believed that there are families who want to keep the patients alive in order to collect the patients' pension. As Informant No.17 stated, "Some families have financial gain in receiving the patients' pension. When I have a family who rarely visits their hospitalized elderly family member, I suspect they are happy as long as they collect the patient's pension during the prolonged hospital stay."

The role of religion in end-of-life decision-making is ambiguous in Japan. Most Japanese answer they have no religion when they are asked religious affiliation, although there are many Buddhism temples and Shinto shrines across the nation. To the best of our knowledge, no researcher has so far elaborated the situation in an appropriate and convincing manner. In this study, only one informant mentioned some influence from a Buddhist tradition in which small portions of food are often offered to deceased ancestors enshrined in a family altar at home. The informant No.28 stated, "It is simply natural to provide food to everybody, regardless of their physical condition, because we offer food even to the dead in Japan."

#### Reimbursement-related factors

To shorten the length of in-patient stays, acute care hospitals want to smooth the process of discharging patients to long-term care facilities. According to informants at acute care hospitals, it is common practice to begin PEG feeding for patients who cannot eat and have lost decision-making capacity before they are transferred to long-term care hospitals. This practice is at the request of physicians at long-term care hospitals, who prefer PEG tubes to nasogastric (NG) tubes. As informant No.22 stated, "Some long-term care hospitals don't accept patients who are unable to be fed by mouth unless a PEG feeding tube is put in place first." In addition, increasing reimbursement rates for PEG make the procedure more attractive for hospital operators, according to some informants. The reimbursement price for PEG increased from 64,000 yen (~ US$550) to 75,700 yen (~US$660) per procedure in 2000, followed by a further increase to 94,600 yen (~ US$820) in 2002. Aside from this, the per-diem payment system at long-term care hospitals makes intravenous hyperalimentation (IVH) financially unfavorable.

Long-term IVH administration for elderly inpatients used to be a standard procedure even for those with normal digestive function at a time when fee-for-service reimbursement system was universally applied in Japan, and reimbursement rates for IVH were high. With the introduction of the inclusive per-diem payment system, costly IVH administration became financially unfavorable. IVH administration for long-stay inpatients has now been replaced by PEG tube feeding, not because the latter is superior to the former based on medical evidence, but because the former has been made financially unattractive under the new system. As informant No.12 explained, "If an IVH solution worth 8,000 yen (~US$70) a day is administered for two weeks at an acute care hospital, the hospital gets this amount reimbursed. But this cost would not be covered by the inclusive per-diem payment system at a long-term care hospital like ours. That's why we have no choice but refuse patients who need long-term IVH."

### Informing families of the use of PEG as a decision, not as an option

Given the circumstances discussed thus far, many physicians decide to perform PEG on a patient before they explain the procedure to the family. When obtaining "informed consent" to perform the procedure from the family, many of the physicians are aware that the "informed consent" is not appropriate technically, because they have led the family to agree with the physician's decision. As Informant No.3 stated, "I tell the family, 'You'd better decide on performing PEG now, otherwise the patient will die'. If I want to lead the family in a certain direction, I have no difficulty in doing this". Some physicians describe overt paternalism. As Informant No.2 stated, "I tell the family, 'We will do it (perform PEG)' when the patient is unable to eat.' It is not a way of offering an option, but just telling it as the next step. There is almost no consideration for patients' preferences toward the procedure. We don't actually take the family's opinion into account, either. "

### Physicians who offer the option of withholding or providing ANH

The small number of physicians who did present this option, in contrast, believed it should be the decision of the family and not of the physician. The theoretical sampling of informants identified 4 physicians (No.11, No.13, No.25, and No.30) in this category. Three were working at long-term care hospitals and one was working at a hospital with both acute care and long-term care wards. These 4 physicians raised the option of withholding ANH after spoon-feeding became difficult in patients with dementia in the long-term care hospitals. These physicians shared several characteristics. First, compared to the majority group, they spent considerable time communicating about the patient's end-of-life care with the family. Informants reported that with repeated consultation they could establish a rapport with the family, which in turn made them feel that the risk of litigation was decreased, although not eliminated. Informant No.30 stated, "I used to be concerned about possible legal problems....But now, I do not worry about this, because decisions regarding the patient's end-of-life are made after much discussion with the family. I believe this decision-making process will avoid a legal problem. In my opinion, a legal problem would arise when communication between the physician and family is insufficient." These informants also consulted with all of the other staff in charge of the patients about whether to withhold ANH, and felt that the team-based approach further reduced the risk of litigation and prosecution. In contrast, some of the majority group of physicians expressed concerns over possible "whistle-blowers." As Informant No. 20 stated, "If I withheld ANH, nurses will secretly tell it to the police or mass media. I definitely think so."

Informants who offered families the option of withholding ANH also provided information about possible prognoses with and without this treatment, as well as the burdens and benefits of each feeding method. Informants who routinely provided ANH tended to report that they did not discuss possible prognoses or alternative feeding methods as mentioned above.

Most families who were offered the option with full information by these 4 physicians decided to withhold ANH. As Informant No.13 stated, "PEG was performed on 2 out of 23 potential ANH patients last year at my hospital."

The minority group of physicians also stated that they gave small amounts of fluid either intravenously or hypodermically to those who did not receive ANH. One of the physicians said the small amount of artificial hydration is helpful to both the healthcare team and patient's family in reducing the psychological burden resulting from 'doing nothing.' Informant No.25 stated, "300 cc of intravenous fluid administration is never enough to sustain life...But it would be painful for both family and the healthcare team to do nothing and stay by the patient's bedside observing the patient waning day by day. Therefore, we give the patient 300 cc of fluids a day as a sort of halfway measure. The small amount of artificial fluid administration helps us overcome the image of 'doing nothing.' This is indeed helpful."

These physicians also recognized that the advantages of PEG tube feeding in this patient group may be balanced by the detrimental. Ease of placement, for example, allows physicians to make an easy decision to perform PEG. Informant No.30 stated, "I used to think that PEG was a really good method, because PEG tube feeding causes much less discomfort than NG tube feeding. In addition, PEG can be completed within 10 minutes. But later, I questioned anew if it was really good, whether the patient and family really wanted it, when considering a prolonged dying process or possibly worse dying process troubled by complications caused by PEG".

The minority group of physicians also shared the viewpoint that ANH is not indispensable in patients believed by the physicians to have entered the last stage of life. Unlike the majority, these physicians said that withholding ANH from dying patients leads to a peaceful death with little suffering, not a painful one. Informant No.13 stated, "Patients have passed away peacefully without ANH. So far, I have observed no suffering in the patients. It is far from the image of 'death by starvation.' At my hospital, not only doctors, but nurses and care workers have accepted this way of dealing with the end-of-life of elderly patients with end-stage dementia".

### Many physicians do not want PEG for themselves

Asked whether they would want PEG tube feeding for themselves if they were in the situations faced by their patients, 16 of the 30 informants stated that they would not want it or would flatly refuse it, while 4 said they personally did not want it, but they would accept it if their family wanted them to do so. Demonstrating the family-oriented decision making, Informant No.29 said, "I have told my wife I don't want tube feeding at the end of my life. But she has said she wants me to stay alive as long as possible. So I would accept it if it could make her feel better." One informant (No.20) said that he would accept it, but only so that the physician in charge would not be prosecuted. The informant stated, "I don't want to invite a situation in which the physician in charge could be prosecuted under the legal framework in this country." Of the remaining 9 informants, 5 said that they would leave the matter entirely to their families, 3 said they did not know, and only one physician would want to have it, because he believed that 'death by starvation' would cause pain and suffering. Informant No.1, reflecting the majority view, stated, "I would absolutely refuse PEG tube feeding. I am sure none of the medical or nursing staff at my hospital would want it for themselves. Well, we should give much thought as to why we give something to our patients that we would never want for ourselves." Asked why they provide their patients with a treatment they would not want for themselves, Informant No.17 stated after a moment, "Well, it is certainly a contradiction. I have never thought of this question before."

## Discussion

The main findings of this study suggest that the nation's medical-legal system directly or indirectly promotes the routine provision of ANH through PEG tube feeding for older patients with severe cognitive impairment. Lack of relevant legislation or guidelines that protect physicians when withholding and withdrawing life-prolonging medical treatment in Japan [29] creates a strong incentive for many physicians to provide ANH irrespective of patients' conditions. It is believed that the physicians' concern over possible legal problems has increased at a time when some physicians who had withdrawn mechanical ventilation from dying patients have been under police investigation on suspicion of murder in Japan [29]. Besides, the number of lawsuits filed against physicians has been on the rise in this country, about 1,000 cases in 2006 across the nation, which is double the rate from 10 years earlier [30]. Many physician informants said, however, that they would be unwilling to be provided with this feeding method if they were in the same situation as their patients. Of the 16 informants who would refuse ANH for themselves, 13 routinely provide the treatment to their patients. A few informants feel perplexed as to why they provide patients with a treatment they would not want for themselves.

Physicians also provide ANH for this patient group partly to maintain the peace of mind of families, and their own peace of mind, by avoiding 'death by starvation.' Most physician informants thought they had no choice but to give ANH for every patient who is unable to take food or fluids orally. This climate of 'inevitability' has been reported in the U.S. as well [31].

A number of physicians stated that almost no individual patient preferences were considered when deciding to perform PEG. This seems partly because the Japanese cultural environment is favorable for physicians' paternalism [32], and also partly because physicians have virtually no way of determining the preferences of formerly competent patients, as there is no widespread, effective policy in place for this purpose in Japan. The lack of advance directives has also been found associated with a greater likelihood of feeding tube use in the U.S. [10].

Most of the physician informants provided what they believed was a life-prolonging measure in the hope of meeting the expectations of families who, the physicians believed, wanted to keep the patients alive. The physicians' intentions, however, may have failed to serve the actual desires of the families in cases where end-of-life communication between the physicians and the families was insufficient.

This study also found a small number of physicians who offer patients' families the option of withholding, as well as that of providing, ANH for patients with advanced dementia. This minority group of physicians shares certain characteristics that were found to reduce some of the effects of the PEG-promoting factors. They believe they reduce the legal risks by making individual efforts to communicate carefully with the patient's family. Based on their subjective judgment, they also believe that they would not be challenged in court, because they have fostered a relationship with the family based on trust. Aside from that, they are also not afraid of possible in-house "whistle blowers" as they have taken a team-based approach to treat patients. Another finding to note is that this minority group of physicians does not believe ANH is indispensable for any patient who cannot take food or fluids orally. Their view is in line with a body of literature which indicates that neither the withholding or withdrawing of ANH from debilitated patients result in gruesome, cruel, or violent death, but leads to a peaceful death with little suffering [33-38].

Instead of tube feeding, these informants give patients small amounts of fluid intravenously or hypodermically as a sort of halfway measure, mainly serving the purpose to make care providers and family overcome the image of "doing nothing." Although potentially controversial, this practice has also been reported in Canada as well [39]. Overall, we believe the alternative practice is more appropriate than the practice of the majority group of physicians when dealing with these patients in light of a number of previous studies that demonstrated that tube feeding provides no significant clinical benefit for these patients [3-8].

The findings of this study indicate that financial incentives may also play a role in promoting PEG tube feeding among cognitively impaired older adults, as previously reported in the U.S. [40]. In an effort to promote better care and more rational use of resources in the final stages of dementia, consideration should be given to adjusting reimbursement policies [40]. The issue of ANH through PEG tube is expected to become much more serious in Japan at a time when the nation, which has the world's greatest longevity, continues to age, and the sale of PEG kits has been steeply increasing from 6,500 in 1993 to 110,010 in 2005 [41].

### Limitations

A major limitation of this study is that the findings were based solely on interviews with physicians; families' preferences and feelings discussed in this study were from the informants' point of view. To obtain a broader understanding, qualitative research with families who have relevant experience will be necessary. The generalizability of our results is limited because of the qualitative methodology, although the number of informants is not small for a study using Grounded Theory approach. Another point to note is that this article deals with the end-of-life issue, independent of any religious perspective, which we believe may be surprising to religious people in the world. The vast majority of Japanese people tend to be highly secular, and therefore it is hard to learn how religious beliefs, if any, influence their end-of-life decisions. An analysis of how religious beliefs may influence the topics discussed in this study should therefore be explored by a new study in the future.

## Conclusion

The findings of this study indicate that, in the absence of relevant legislation or guidelines, the framework of Japan's current medical-legal system implicitly provides many physicians with a strong incentive to offer ANH routinely to severely cognitively impaired older patients. Japanese physicians face legal, emotional and cultural barriers when withholding ANH as a life-prolonging measure. This situation is different in some western countries, where legal and cultural attitudes are more accepting of decisions not to treat. Despite the barriers in Japan, this study found that there is a small number of physicians who routinely offer families the choice of withholding as well as providing ANH. They take the legal risks based on their professional belief that the family should be given the choice after acknowledging the patient's prognosis. These physicians try to reduce the legal risks by making individual efforts that focus on communication with the family and staff. They are also less affected by the emotional barriers, the most influential of which is related to the perception of ANH. It seems apparent that end-of-life education should be provided to medical providers to change the widespread provision of ANH in Japan.

## Competing interests

The authors declare that they have no competing interests.

## Authors' contributions

KA was involved in all aspects of the research, including study design, informant recruitment, making interviews, data analysis, and manuscript preparation. MT's role in the study included assistance with study design, informant recruitment, and data analysis and interpretation. HM's role included assistance with data analysis and interpretation. IK's role in the study included assistance with study design, supervision of data collection, data analysis, and preparation of the manuscript. TEF's role included assistance with data analysis and interpretation, and preparation of the manuscript.

## Pre-publication history

The pre-publication history for this paper can be accessed here:


